# The influence of network topology on synchrony and oscillations in networks of spiking neurons

**DOI:** 10.1186/1471-2202-12-S1-P44

**Published:** 2011-07-18

**Authors:** Duane Q Nykamp, Alex Roxin, Albert Compte

**Affiliations:** 1 School of Mathematics, University of Minnesota, Minneapolis, MN 55455, USA; 2 Institut d'Investigacions Biomèdiques August Pi i Sunyer (IDIBAPS), Barcelona, Spain

## 

**Figure 1:**
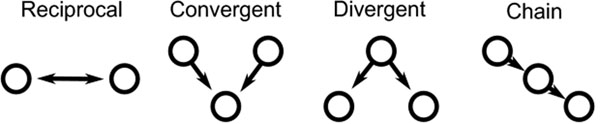
The four second order edge motifs of reciprocal, convergent, divergent, and causal connections.

We use the recently developed framework of *second order networks*[1] to study the influence of network topology on synchrony and oscillations in networks of integrate-and-fire neurons. Second order networks are generalizations of Erdős–Rényi random networks [2], where one can specify the second order statistics among the network connections, i.e., the relative frequency of second order edge motifs that are embedded in the larger network. The second order edge motifs are the reciprocal, convergent, divergent, and chain connections diagrammed in Figure 1. Using the small number of parameters of the second order network framework, one can manipulate global network statistics such as the variance of the in- and out-degree distributions (through convergent and divergent connections) as well as the covariance between in- and out-degree (through chain connections).

Our previous work has identified motifs involving excitatory neurons that strongly influence the dynamical state of the network. In recurrent excitatory networks where the mean current caused neurons to fire, increasing chain connections led to higher network synchrony while increasing convergent connections decreased synchrony [1]. In networks of excitatory and inhibitory neurons where neuron firing was due to fluctuations of the membrane potential (the fluctuation-driven regime), broadening the excitatory incoming degree distribution onto excitatory neurons led to increased oscillations [3]. Since incoming degree distribution is tightly linked with convergent connections, this latter result demonstrated a strong effect of excitatory convergent connections on the network state. In both studies, the divergent connection motif (or the common input motif) did not have a large qualitative influence on the dynamics.

In the present study, we systematically investigate the influence of second order network motifs on the dynamical state of recurrent networks of excitatory and inhibitory neurons in the fluctuation-driven regime. We simulate networks of sparsely coupled integrate-and-fire neurons, where the connection probabilities are given by the second order network model. We allow the probability of each second order motif to depend on the populations (excitatory versus inhibitory) of the neurons involved in the motif. We again find that divergent connections alone have little qualitative effect on the dynamics. However, both convergent connections and chain connections dramatically affect the onset and magnitude of synchronous network oscillations, either increasing or decreasing oscillations depending on the populations of the neurons in the motif. We explain these results through analysis of a mean-field model of the coupled populations.
